# The effectiveness of Tai Chi for patients with Parkinson’s disease: study protocol for a randomized controlled trial

**DOI:** 10.1186/s13063-015-0639-8

**Published:** 2015-03-24

**Authors:** Yan Yang, Yan-lei Hao, Wen-jing Tian, Li Gong, Kui Zhang, Qi-guang Shi, Da-fang Sun, Cui-lan Li, Zhi-ling Zhao

**Affiliations:** Department of Neurology, Affiliated Hospital of Jining Medical University, 79 Guhuai Rd, Jining, Shandong China

**Keywords:** Parkinson’s disease, Tai Chi, Exercise, Balance, Gait, Quality of life

## Abstract

**Background:**

Parkinson’s disease (PD) is a common degenerative neurological disorder that causes loss of independence and decreased quality of life. The prevalence of PD tends to increase with age. In China, the morbidity rate of PD among people aged more than 65 years old is 1.70%. As an important component of traditional Chinese *Qigong* exercises, Tai Chi is a popular and safe exercise, especially for older adults in China. And it may result in promising gains for PD patients. However, current evidence is insufficient to inform the use of Tai Chi in the management of PD. Therefore, the aim of this trial is to systematically evaluate the effect of Tai Chi on PD and determine whether Tai Chi is an eligible exercise program for Chinese PD patients.

**Methods/Design:**

A single-blind, parallel randomized controlled trial will be conducted. One hundred and forty-two patients with PD will be randomly assigned to a Tai Chi group (n = 71) or routine exercise group (n = 71). Subjects will participate in supervised study programs 3 times per week for 2 months and will be followed for an additional 6 months after formal training stops. The primary outcome measures include Berg Balance Scale, Timed Up and Go Test and Six-Minute Walk Test, which are known to be valid and reliable clinical instruments. The Unified Parkinson’s Disease Rating Scale Motor Section and Parkinson’s Disease Questionnaire-39 will be used as the secondary outcome measure. All outcomes will be measured at baseline, 2 and 8 months. The sample for this trial (N = 142) will provide relevant information to detect the improvement of balance, gait and quality of life in either of the 2 exercise groups.

**Discussion:**

Findings from this study will provide insights into the effects of Tai Chi in people with PD. The information gained from this project has the potential to influence the clinical decisions of Chinese doctors, and will provide clear evidence as to whether Tai Chi should be advocated in people with PD.

**Trial registration:**

The trial was registered at (ChiCTR-TRC-14004549) on 22 April 2014.

## Background

Parkinson’s disease (PD) is a common neurodegenerative disorder that is associated with four landmark symptoms of resting tremor, bradykinesis, rigidity and decreased postural reflexes [1]. Globally, there are at least 4 million people estimated to be diagnosed with PD [2]. Although the specific causes of PD are unknown, incidence increases with age, especially after 50 years old [3]. In China, PD prevalence is 1.70% in people aged more than 65 years old [4]. It is reported that in people with PD it is common to develop a slow, shuffling and dysrhythmic gait [5]. Also, most people with PD will ultimately develop impaired balance [6]. Despite advances in medical care, gait disturbance and balance decrements worsen as the disease progresses, contributing to loss of independence, falls and poor quality of life [7]. Therefore, people with PD gradually place a strain on societies, healthcare and financial systems.

Exercise, as one of non-pharmacological interventions in assisting the treatment of PD, has demonstrated efficacy in improving gait disturbance, balance decrements and frequency of falls in patients with PD [8-10]. It may improve brain health by suppressing oxidative stress, stabilizing calcium homeostasis and plasticity-related changes in the central nervous system including enhanced glucose utilization, synaptogenesis, angiogenesis and neurogenesis [11-14]. Also, there is supporting evidence indicating that intensive exercise is more effective in controlling balance and gait for PD in relevant studies [15]. However, the prevalence of older Chinese people using such exercises is quite low. As an important component of traditional Chinese *Qigong* exercises, Tai Chi is an ancient art and science of healthcare that is quite popular with Chinese older adults.

Tai Chi, as a mind-body exercise, consists of a series of dance-like movements linked in a continuous sequence, flowing slowly and smoothly from one movement to another that emphasizes weight transfer and movement of the body [16]. By doing so, it may improve postural control that can also benefit balance, gait and quality of life and some studies have demonstrated the beneficial effects of Tai Chi in improving gait and balance [16-18]. The last systematic review concluded that Tai Chi resulted in promising gains in mobility and balance among PD patients, but they were significantly limited due to small sample size, short follow-up period, poor methodological quality and heterogeneity in population characteristics with few Chinese PD patients being included [19,20]. Thus, new and high-quality randomized controlled trials are warranted to prove the previous findings, especially for Chinese PD patients.

The aim of this randomized controlled trial is to systematically evaluate the effect of Tai Chi in improving gait, balance and quality of life in patients with PD in China by observing the difference between the Tai Chi group and routine exercise group. The hypothesis is that patients with PD who are treated with Tai Chi will experience a greater reduction in Parkinsonian symptoms as well as improved gait, balance and quality of life in comparison with those treated with routine exercise.

## Methods and Design

### Trial design

A single-blind (assessors), parallel randomized controlled trial will be conducted to compare Tai Chi with routine exercise (allocation ratio 1:1). The study period is 8 months including a 2-month supervised intervention and a 6-month follow-up with the primary outcome measured at baseline, 2 and 8 months. The primary site for conduct of the study, including all study visits and Tai Chi interventions will be conducted at the Affiliated Hospital of Jining Medical University, Jining, Shandong, China. Routine exercise will be conducted in a home-based environment.

### Participants and recruitment

People attending Medicine for the Elderly or Neurology outpatient appointments at Affiliated Hospital of Jining Medical University, Jining, Shandong, China, for diagnosed or probable PD will be invited to participate in the study.

### Inclusion criteria

1. Adults diagnosed with, or with probable idiopathic, PD with a disease severity rating of stage 1 to 4 on the Hoehn and Yahr scale (which ranges from 1 to 5, with higher scores indicating more severe disease) [21].
2. Prescribed one or more anti-parkinsonian medications by a consultant neurologist or consultant physician with specialist knowledge of neurological disorders.
3. Chinese speaking and literate (participants are required to actively engage in the therapy process).
4. Stable medication use; that is, not altered within the previous month and not expected to change during the period of the research project (8 months).
5. Without dementia or significant cognitive impairment. The clinical team will judge whether the patient has the cognitive capacity required to participate in the trial; that is, has read patient information, complete self-report questionnaires and engaged actively in the intervention process.
6. Willing to be assigned to any of the two interventions.

### Exclusion criteria

1. Suspected Parkinsonism due to causes other than idiopathic PD.
2. Current participation in any other behavioral or pharmacologic study or instructor-led exercise programs.
3. Participation of a structured exercise program in the preceding 2 months.
4. Unavailability during the period of the study.

### Recruitment

**Figure 1:**
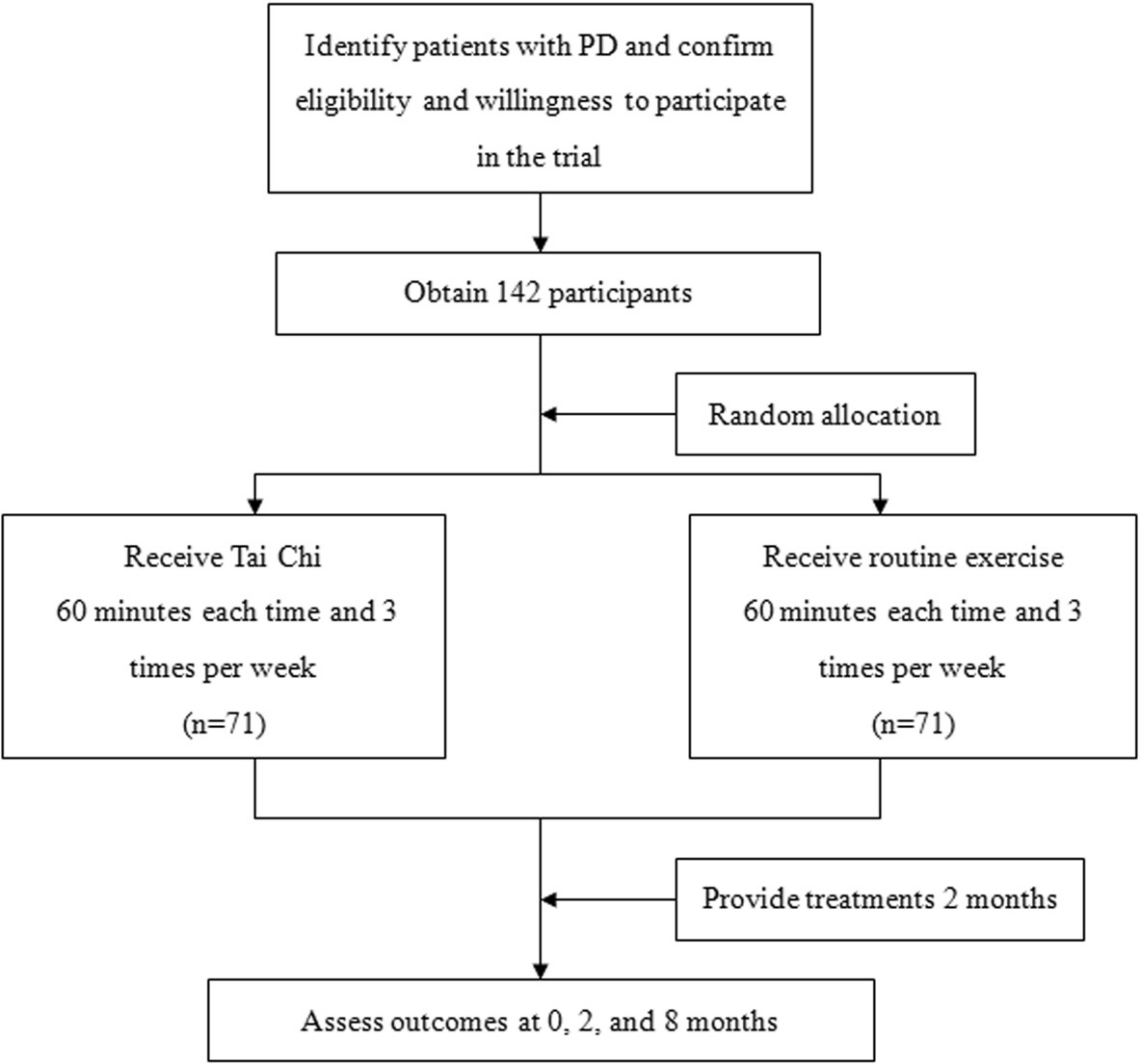
**Study design, recruitment, randomization to treatment, and outcomes assessment.** PD: Parkinson’s disease.

Based on inclusion criteria 1, 2, 3, 4 and 5, participant identification will be performed by the clinical team 2 weeks prior to upcoming outpatient appointments. An information package containing: a patient invitation letter, participant information sheet, and a consent form will be posted out to potentially eligible participants. The patient information will be supplemented by an information sheet for the spouse/carer. Patients will be required to return the participant information sheet and the accompanying consent form. Patients who did not return the materials will be contacted by telephone to discuss their suitability for participation. Patients who are willing to participate but do not meet eligibility criteria will be thanked for their interest. Eligible patients will be approached for their consent to participate in the trial on the day they attend clinic for their outpatient appointment. If the consent is obtained, the patient would be recruited and the whole recruitment will be achieved by a 10- to 12-month rolling program (Figure 1).

### Randomization

After completing the baseline test, each participant will learn his/her group assignment by receiving a sealed, sequentially-numbered envelope from the project director and opening the envelope that contains the group assignment randomly assigned to his/her sequence number. The sequence numbers are generated by using Statistical Package for the Social Sciences (SPSS) 18.0 statistical software (SPSS Inc., Chicago, IL, USA). Measurement technicians who are responsible for data collection are blinded to participant group assignment.

### Outcome measures

#### Primary outcome measures

All participants will be measured before commencing each intervention (pre-test) and upon completion (post-test). The primary outcome measure includes the Berg Balance Scale (BBS), Timed Up and Go Test (TUG) and Six-Minute Walk Test (6MWT), which are known to be valid and reliable clinical instruments [22]. According to the wide disability spectrum associated with PD, balance impairments are common in patients [23]. The BBS will be utilized to identify potential training effects on balance [24]. Functional gait performance will be measured by the TUG [25]. Participants will be asked to walk as quickly as they safely can under all conditions and will be permitted to use their usual gait aid. The TUG measures a participant’s ability to perform sequential gait movements and has been proved to be a reliable outcome measure for use in PD. In addition, the 6MWT will be completed to quantify walking capacity, measured as the distance covered in which conducted on a 20-meter walkway next to the laboratory and we record total distance traveled. This test has demonstrated high reliability in people with PD [26]. Assessment will be performed during the patient’s self-reported optimal ‘ON’ period, often 1 hour after medication.

#### Secondary outcome measures

The Unified Parkinson’s Disease Rating Scale Motor Section (UPDRS-III) [27] and the Parkinson’s Disease Questionnaire-39 (PDQ-39) will be used as the secondary outcome measures. The UPDRS-III will be administered by a trained clinician who is blinded to group assignment. Health-related quality of life will be measured using the PDQ-39, a generic health-related quality of life measure, which is specific to the PD population [28]. Participants will also be assessed at peak dose (approximately 1 hour post administration) of anti-Parkinsonian medication. Several measures will be taken to characterize participants at each assessment session. Severity of PD will be categorized with the Hoehn and Yahr scale [21] and freezing will be characterized by the Freezing of Gait Questionnaire [29], confidence will be measured by the Ambulatory Self-confidence Questionnaire [30]. General demographic information to be recorded will be: age, disease duration, medical history, mobility aid use, medication type and dosage, and so on. Medication will be documented in detail at every assessment time point.

### Intervention

#### Tai Chi

The intervention will be composed of 1-hour group Tai Chi classes customized for patients with PD, and held 3 times weekly for 2 months. The program will include traditional warm-up exercises, followed by 6 simplified Tai Chi movements taught by 2 certified and experienced instructors (average experience of 20 years). Warm-up exercises (15 minutes) will include weight-shifting, arm-swinging, gentle stretches of the neck, shoulders, spine, arms, legs, visualization techniques, and traditional breathing methods (that is whole-body breathing). These exercises will help release tension in the physical body, incorporate mindfulness and imagery into movement, increase awareness of breathing, and promote overall relaxation of body and mind. The core Tai Chi movements (45 minutes) will be adapted from the Yang Short-form style of Master Cheng Man-ching [16], and performed repetitively. The six movements are as follows: (1) wave hands like clouds; (2) part the wild horse’s mane on both sides; (3) step up and thrust downward; (4) strike to ears with both fists; (5) repulse monkey; (6) grasp sparrow’s tail. These movements were selected because they are easy to comprehend and emphasize repeated bilateral stepping with body-weight transfer that is helpful to retain postural stability for PD patients. Chairs will be provided for resting, and patients will be allowed to progress at their own comfort and pace. After 2 months of training, the Tai Chi group will be provided with a 30-minute instructional videotape that outlines the exercises presented in class. Patients will be required to continue to practice at home at least three times per week. Class attendance will be monitored, and adherence to practice will be tracked via self-report logs, which obtain the weekly frequency and duration of home Tai Chi practice.

### Routine exercise

Subjects allocated to the control group will not receive any specific training from the study schedule. They will be requested to keep their original habit of routine exercise (except Tai Chi) such as walking, dancing, cycling, and so on, and they will also be requested to practice for 1 hour at a time, 3 times a week in the training period as well as the follow-up. The follow-up training for both groups will be monitored by phone call.

### Assessment procedures

Data from patients and caregivers will be collected at baseline (T0), 2 months (T1) and 8 months (T2) by 2 research assistants. Another 3 selected and trained occupational therapists will score the BBS, TUG, 6MWT, UPDRS-III and PDQ-39 in an activity that is video- recorded by the assessor. The data collected from the assistants and therapists will be entered in a dedicated computer and kept in a secure and lock-protected location. All assessors and scorers are blinded for group allocation and each participant will be followed up by the same assessor. To test the quality of home-based routine exercise, patients and caregivers will fill in all self-report records. Observational tests or measures that follow a semi-structured interview format will be conducted in the patient’s home environment by the assessor. Considering possible response fluctuations in PD, measures will be administered 1 hour after medication intake (the ‘ON’ phase). For budgetary reasons, the 6-month follow-up training will be monitored by phone call and the data for assessment will be finally collected by the same assessor.

### Statistical analysis

#### Baseline analyses

The assessment of demographic characteristics of participants at baseline will compare the participants who are subsequently randomized and those who are screened but not randomized. Exclusion from randomization will be tabulated by a specific criterion.

### Efficacy analysis of outcome measures

The primary analysis will compare the changes on BBS from baseline to endpoint of treatment between two treatment groups using analysis of covariance (ANCOVA) adjusting by the BBS baseline value. The statistically significant level of *P*-value is less than 0.05. If any participants drop out from the trial, intention-to-treat analyses will be adopted. Participants who withdraw will be treated as having no change from baseline at all times after dropping out. In addition to intention-to-treat analyses, multiple imputation will be used to perform secondary analyses. We will also check the mechanism of the participation drop-out and determine whether the missing data is ignorable or not. If non-ignorable missing data methods are needed, we will explore both selection and pattern-mixture types. All efficacy analyses of primary and secondary outcome measurements will be conducted by the same analytical strategy.

All participants will undergo sub-group analysis test for potential effect modification for the presence of a spouse/carer on the therapeutic effect. Appropriate adjustments will be made in the statistical analyses for potential factors which are unevenly distributed between the two groups. Participants with significantly impairment of balance, gait and quality of life at baseline will be analyzed to determine the independent impact of these disturbances on the therapeutic effect. Adjusted estimates will be obtained by identifying baseline imbalances and incorporating them into a regression model by pre- and post-intervention primary outcome scores. If normal distribution assumptions were not met, non-parametric analysis will be adopted even after suitable transformations.

### Follow-up evaluation

Physical activities and other leisure time activities will be measured during the 6 months follow-up via telephone interviews. This is to monitor changes among participants in activities that can enhance performance on tests, apart from Tai Chi and routine exercise. The self-completed quality of life questionnaire will be mailed to participants after 2 months from randomization. All baseline measurements will be repeated at 6-month follow-up. Researchers performing all measurements will be blinded to group allocation and participants will be required not to reveal to researchers which group they were in (Table 1).

**Table 1:** **Schedule for data collection; outcome measures per visits**

**Measures**	**Baseline (0-week) (T0)**	**End of treatment (8-week) (T1)**	**The first follow-up (24-week) (T2)**
Demographic characteristics	×		
PD history	×		
BBS	×	×	×
TUG	×	×	×
6WMT	×	×	×
UPDRS-III	×	×	×
PDQ-39	×	×	×
Adverse experiences^a^		×	×
Combined medication^b^		×	×

^a^Adverse experiences: any adverse experiences at any visit during treatment sessions and 24 weeks will be monitored. The research team will review all trial protocols, monitor patient safety and investigate any adverse events. ^b^Patients will be asked whether they have used other medication during the treatment. If they have used concomitant medications then the type and dose of medication taken by them will also be recorded in detail.

*Abbreviations:* 6MWT, Six-Minute Walk Test; BBS, Berg Balance Scale; PD, Parkinson’s disease; PDQ-39, Parkinson’s Disease Questionnaire-39; TUG, Timed Up and Go Test; UPDRS-III, Unified Parkinson’s Disease Rating Scale Motor Section.

### Sample size

The sample size was calculated based on a pilot study (not published), including 10 patients with PD (1 to 4 on the Hoehn and Yahr scale) who participated in the Tai Chi exercise. To detect a difference of 4.0 in BBS and assume a standard deviation of 7.2, the sample size of 116 was required at 80% power and 2-sided significance alpha level of 0.05. According to the previous study, the significant difference between Tai Chi and stretching training was reported in TUG of patients with PD based on a sample size of 65 in each group [16]. Therefore, the optimal simple size, in anticipation of a 20% drop-out, would be 71 per group (total 142).

### Ethics

The study has been approved by the Medical Ethics Committee of Affiliated Hospital of Jining Medical University (number 20140327). The investigator will explain the benefits and risks of participation in the study to each subject and will provide an informed consent form approved by the Ethics Committee. Only patients who sign the form will be included in the study. Results will be published anonymously. If there is an unexpected worsening of motor or neuropsychiatric symptoms, patients can immediately contact our clinic 24 hours a day and will be immediately referred to a neurologist specializing in PD.

## Discussion

It is important to realize that the role of exercise should not be considered a cure for the disorder but rather a strategy for maintaining as much normal function as possible. Assessing balance, gait, aerobic fitness, flexibility, agility, coordination, strength and quality of life has become a challenge for fitness professionals working with older adults and those with PD. Currently, there are no established exercise guidelines to appropriately evaluate these factors.

This trial will be the first to compare the effect of Tai Chi training and routine exercise in people with PD in mainland China. Presently, Tai Chi is frequently used as a daily health cultivation exercise in the Chinese population, especially for the older people; although it has been used to test its effectiveness in some diseases such as insomnia, low back pain and even stress, it is, however, not clear whether Tai Chi should be advocated in Chinese patients with PD. Recently, the beneficial role of Tai Chi on PD has been reported in some countries; however, most of them just focus on the recovery of motor function and there is a lack of reliable evidence of follow-up effect for Tai Chi. The best type of exercise to help the PD population likely depends on which best addresses the efficiency of treatment. This trial will reveal the effect of Tai Chi, not only on focusing on balance and gait performance, but also on health-related quality of life, and for a follow-up period of 6 months. This data will assist in investigating the impact of Tai Chi for people with PD who are still mobile and living in their own homes. There are also some limitations in our trial. This is a single-center study and the number of participants included in our study is not large (a total of 142 patients was planned to be recruited). Moreover, the lack of patient blinding may increase bias.

Tai Chi is a traditional Chinese mind-body exercise, continuous in sequence, deep in breath and slow and smooth from one movement to another. It is moderate in exercise intensity. A recent review has reported that moderate intensity exercise may be a reasonable choice for people with PD [31]. Findings from this study will provide insights for Chinese practitioners into the effects of Tai Chi in people with PD. The information gained from this project has the potential to influence the clinical decisions of Chinese doctors, and will provide clear evidence as to whether Tai Chi should be advocated in people with PD. The results of the completed study will be reported according to the Consolidation of Standards for Reporting Trials guidelines [32].

## Trial status

The study has been initiated as planned in June 2014. Recruitment is ongoing. The study will be completed in June 2017.

## Abbreviations

ANCOVA: analysis of covariance
BBS: Berg Balance Scale
6MWT: Six-Minute Walk Test
PD: Parkinson’s disease
PDQ-39: Parkinson’s Disease Questionnaire-39
SSPS: Statistical Package for the Social Sciences
TUG: Timed Up and Go Test
UPDRS-III: Unified Parkinson’s Disease Rating Scale Motor Section
